# Pooling samples for “top-down” molecular exposomics research: the methodology

**DOI:** 10.1186/1476-069X-13-8

**Published:** 2014-02-13

**Authors:** Heqing Shen, Weipan Xu, Siyuan Peng, Hagen Scherb, Jianwen She, Kristina Voigt, Ambreen Alamdar, Karl-Werner Schramm

**Affiliations:** 1 Key Laboratory of Urban Environment and Health, Institute of Urban Environment, Chinese Academy of Sciences, 1799 Jimei Road, Xiamen 361021, PR China; 2 Helmholtz Zentrum München - German Research Center for Environmental Health (GmbH), Institute of Computational Biology, Ingolstädter Landstr. 1, 85765 Neuherberg, Germany; 3 Environmental Health Laboratory Branch, California Department of Public Health, Richmond, CA 94804, USA; 4 Helmholtz Zentrum München - German Research Center for Environmental Health (GmbH), Molecular EXposomics (MEX), Ingolstädter Landstr. 1, 85764 Neuherberg, Germany; 5 Department für Biowissenschaftliche Grundlagen, Technische Universität München, Weihenstephaner Steig 23, 85350 Freising, Germany

**Keywords:** Exposome, Exposure biomarker, Metabolome, Effect biomarker, Case-control study, Environmental pollutants, Pooled sample

## Abstract

**Background:**

Exposomics is the cutting-edge concept of screening the environmental risk factors for disease. In the novel “top-down” approach, we estimate the molecular exposome by measuring all body fluid analytes in a case-controlled study. However, to detect diverse pollutants, a sufficient sample size and multiple analytical methods are required. This may lead to dramatically increased costs and research workload.

**Methods:**

To help reduce complexity, we suggest a sample pooling strategy along with a scheme for combining both general unknown or multi-targeted screening with targeted analysis. The sample pooling method was tested using computer simulations.

**Results:**

By comprehensively analysis of pooled samples, it is possible to identify environmental risk factors. Factors are initially screened in the pooled case and control population samples, then in the randomized grouped and pooled case and control subpopulation samples. In the sample grouping, five or more pools were suggested for groups having 30 individuals per pool.

**Conclusions:**

This study suggests that sample pooling is a useful strategy for exposomics research, which provides a hypothesis-free method for pollutant risk screening.

## Introduction

An estimated 70 to 90 percent of risk factors associated with chronic diseases are thought to arise from individuals’ exposure to environmental hazards [1-3]. This drastic connection raises interest in analyzing ubiquitous environmental risk factors which collectively constitute the “exposome” [4], a concept first introduced by Wild [6] and later advocated by Rappaport [4,7]. There is particular interest in analyzing the role of chemical pollutants in epidemiological studies [5]. Chemical pollutant exposomics thus provides an approach to systematically assess pollutant risk for defined health outcomes in a given population.

In a so-called “top-down” strategy [4,7] all blood analytes including both small endogenous and exogenous molecules can be clustered into one of three groups: environmental chemical pollutants and their metabolic residuals i.e., chemical pollutants’ exposome, the blood metabolome response to chemical pollutant exposure, and response to other environmental risk factors such as noise, radiation etc. The goal of “top-down” exposomics is to identify biological analytes (i.e., biomarkers) relevant for a defined outcome or disease. Both environmental pollutants and endogenous metabolome need to be considered, although some analytes are sometimes confounded factors in the theory (for example, environmental pollutants and their metabolomic responses in biology).

The methodology discussed in this study majorly focuses on the broad analysis of environmental pollutants and their metabolites in case-control samples. We propose that the chemical pollutant exposome may be screened in a similar way as in disease-oriented metabolomics analysis [8]. Furthermore, it may aid in the development of systematic biological models for assessing and illustrating the risk and metabolic impact associated with the exogenous exposome [9]. Lastly, we show that exposure to arsenic in males is linked to oligozoospermia via analysis of pertinent metabolomic biomarkers [10].

Quantitative assessment of the risks associated to pollutant exposure [7,11] can be challenging. For instance, molecular epidemiology usually requires large sample numbers in order to verify the hypothesized relationships. Furthermore, bio-monitoring of environmental pollutants is sometimes hampered by their diverse nature in necessitating a set of integrated methods [12,13]. In addition, limited sample volume makes multiple measurements on individual samples difficult or impossible. Considered together, the requirement of large sample size and multiple analyses for each sample proves costly and labor intensive when working with human data. To help overcome these challenges, we propose a sample pooling strategy for molecular exposomics, in which fewer, but larger volume pooled samples are analyzed with the tradeoff losses of the signatures from individual observations in the case-control study.

### Chemical oriented exposomics requires an integrated sample matrix

Bio-monitoring analysis following chemical exposure is commonly obtained via chemical-oriented targeted measurements. When xenobiotics enter the body, they either accumulate, or are processed metabolically and excreted. Therefore, analytes employed as biomarkers for monitoring could be either parental compounds, their metabolites or their conjugated derivatives with endogenous molecules via enzymatically catalyzed transformations. Generally, non-polar, lipid-soluble forms of chemicals will be metabolized into polar, water-soluble forms and excreted in bile and urine. It is possible to group target pollutants into four families according to various physicochemical properties, as shown in Table 1[12,13]. These families are:

1. Persistent organic pollutants (POPs), which tend to accumulate in fatty tissues and redistribute among the other parts of the body via blood as a transport vector. These are usually measured by GC-MS (or HRGC-HRMS) with the exception of some perfluorinated compounds.

2. Readily degradable compounds, which can readily transform to their metabolites through phase I reactions and further on partly conjugate with endogenous molecules (e.g., glucuronate, sulfate, mercapturic acid ester, acetyl ester, and so on) through phase II reactions to be easily discharged in urine, bile and sweat. These are normally measured using LC-MS techniques.

3. Accumulated inorganic pollutants, which may be deposited in the kidney (such as cadmium) or bone (such as lead), and which are commonly measured by LC-ICP-MS.

4. Non-accumulating inorganic pollutants such as arsenic, commonly measured by LC-ICP-MS.

**Table 1:** Samples and integration of analytical methods for chemical oriented instrumental measurement

**Sample types**	**Urinary matrix**					**Blood matrix**			
**Species**	Free form	Conjugated form				Protein conjugated form		Free POPs	
**Pollutant**	Metal or metalloid	Mercapturic acid	Glucuronic acid	Sulfate	Acetyl	Metal or metalloid	HSA-Cys34	Lipophilic POPs	Perfluorinated compounds
**Techniques**	ICP-MS	UPLC-HRMS(CNL MS/MS)				ICP-MS	UPLC-HRMS(SRM MS/MS)	GC-SIM-MS	LC-MS/MS
**Analysis approach**	1	2	3	4	5	6	7	8	9

CNL: constant neutral loss; GC: gas chromatography; HAS: human serum albumin; POPs: persistent organic pollutants; HRMS: high resolution mass spectrometer; ICP: inductively coupled plasma; MS: mass spectrometer; SIM: Selected ion monitoring; SRM: Selected reaction monitoring; UPLC: ultra performance liquid chromatography.

Pollutants which are more efficiently metabolized accumulate in urine, and urinary bio-monitoring provides a suitable approach for assessing their internal exposure doses. However, blood samples are still usually preferred for monitoring most persistent pollutants. Although many other types of human samples can be used to ascertain pollutant residues [12,13], blood and urine are the two most viable due to sampling difficulty, analyte enrichment, and sample preparation complexity arising in the bio-monitoring of other tissue samples types.

To systematically assess the chemical pollutants exposome, it is necessary to employ a general, untargeted screening analysis. This can be followed by a targeted analysis based on initial findings from the pre-screening. For example, a screening analysis for pollutants could be addressed by using approaches such as adductomics for characterizing electrophilic chemicals [14], whereas multi-targeted pollutants analysis could run on priority lists suggested by the US EPA [15] or European Commission [16]. Since untargeted screening analysis usually has a worse sensitivity than commonly used targeted analysis, it is necessary to utilize pollutant enriched matrices in order to trace environmental contamination levels. Although blood is suitable for the measurement of a diverse range of pollutants [12,13], pollutant metabolites enriched in urine are usually less abundant than in blood, rendering urine complementary to blood for comprehensive exposome analysis.

### Chemical-orientated exposomics requires an integrated analytical approach

Complementary techniques are also required for screening the diverse chemicals in urine and blood (Table 1). Usually, investigations of the chemical exposome are based on the different types of mass spectrometers [17-19]. For targeted and/or untargeted detection of various trace level compounds, high throughput and sensitive mass spectrometry (MS) techniques, such as high resolution MS (HRMS), time-of-flight MS (TOF-MS), and Orbitrap MS have been employed. At present, the coupling of different chromatographic separations by various MS with different ionization techniques provides the most sensitive and specific platform for coping with the wide variety of molecules present in human tissues. For example, in a common configuration, gas chromatography (GC) is used for the separation of thermally stable, volatile and less polar molecules, while liquid chromatography (LC) is used for the separation of thermally labile, non-volatile, more polar chemicals. Lastly, at least three different ionization sources are typically required: electron impact (EI) for volatile or semi-volatile organic pollutants, electrospray ionization (ESI) for readily ionizable water-soluble polar pollutants, and inductively coupled plasma (ICP) for inorganic pollutants.

To analyze different types of chemicals, multiple analyses of the same samples are also generally required. For instance, POPs such as PCBs and PBDEs can be analyzed by HRGC-HRMS at least 50 μL blood without a complex sample cleanup procedure [20], whereas, another 50 μL blood might be investigated for perfluorinated compounds by LC-ESI-MS [21]. One integration scheme for dealing with systemic measurement is proposed in Table 1. By applying well-developed omics approaches to the task of biomarker mining [22,23] it is possible to extricate risk factors from exposome data.

As discussed earlier, measurements of individual samples in an exposomic study for a large population results not only in an increased analytical workload, but can also rapidly become prohibitively expensive. Additionally, to profile and quantify the chemical exposome in a case-control study, large sample volumes are required from participants (typically blood, urine or both), in order to facilitate multiple measurements. In a typical bio-monitoring study, 2-5 mL of blood and 2-10 mL of spot urine are collected; however such a small sample volume may not be sufficient to screen for all pollutants. Furthermore, collection of larger sample volumes may lead to decreased study participation. The sample pooling strategy suggested in this study is designed to help mitigate these problems.

### Preliminary exposome analysis using pooled population samples

For disease-oriented exposome analysis, we suggest pooling samples separately for case and control populations. Equal fractions of individual samples are mixed from each population separately, and afterwards qualitative and semi-quantitative screening of the composed samples can be conducted. To adjust for potentially confounding factors such as sex, age or race, we also advocate sample stratification before pooling [24]. Sample pooling has the advantage that: (1) sample volumes are large enough for multiple measurements, and (2) analytical workload can be greatly reduced. Large sample volumes allow measurements to be repeated 6-10 times, which provides the power to demonstrate statistical significance of the analytical variation of the mean value [25-27]. In addition, large sample volumes are suitable for a wide range of analytes measurement by using the different methodologies.

However, while pooled samples facilitate the robust analysis of diverse chemical analytes, only population means (*μ*_*case*_ and *μ*_*control*_) are available following such analysis [25-28]. In other words, among the two sources of variation, only the between-measurement analytical variation but the between-subject biological variation is available. So analyte distributions in the case and control samples are forfeited, including the variances of (*σ*^*2*^_*case*_ and *σ*^*2*^_*control*_); therefore, the results compromise the comparison between the case and control [28]. Although less straight-forward than for unpooled samples, one can nonetheless estimate analytical variation by performing at least 6 repeated measurements the blood or urine samples. Importantly, using pooled samples, it is still possible to compute the populations mean ratio (*μ*_*case*_ / *μ*_*control*_) or fold change (FC) between case and control, a common measure for establishing differences in analyte concentration. Furthermore, as with gene microarray analysis and metabolomics analysis, FCs can be used for establishing the references or cut-offs [23]. Because pooled samples are based on diseases, the preliminary pollutant screening may directly link the chemicals to their risks.

### Primary exposome analysis using pooled subpopulation samples

The inter-subpopulation variance analysis of the means of pooled subpopulation samples could augment disease-oriented analyte screening. It stands to reason that if there is a significant difference of an analyte X between case and control populations, there should also be a significant difference in the constitutive subpopulations.

Randomized grouping the individual samples is the fundamental strategy in the preparation of pooled subpopulation samples. According to the general central limit theorem, irrespective of the true distribution of an analyte X in the population, the means of the sampled subpopulations tend to a normal distribution as the number of sample pools increased to five and above [27]. Thus, adequate numbers of samples per pool and adequate numbers of sample pools must be taken into consideration during study design [28]. For example, a population of 300 individuals evenly split into case and control subpopulations can be used to construct 150 case individuals and 150 control samples using 30 samples per pool. In any case, subpopulations are assumed to exhibit the same distribution patterns as the unpooled populations. The means of the pooled samples (i.e., ${\bar{x}}_{\mathrm{case},k}$ and ${\bar{x}}_{\mathrm{control},k}$; where *k* is the pool number) and the inter-subpopulation variances (i.e., $s_{\mathrm{subp},\mathrm{case}}^{2}$ and $s_{\mathrm{subp},\mathrm{control}}^{2}$) can be calculated by using formula (1). The pooled case and control sample means are normallz distributed, and the hypothesis of differing sample mean can be tested by using the t-Test.

(1) $$s_{\mathrm{subp}}^{2}=\frac{1}{k}\sum_{i=1}^{k}{\left(\bar{x_{i}}-\mu \right)}^{2}$$

Since subpopulations’ means and their inter-subpopulation variances can be measured in a cost-efficient manner, the sample pooling technique has been proposed for contaminant biomonitoring [26]. Although statistically significant differences in sample means between case and control can be calculated using pooled samples, statistical sensitivity to detect differences may still decrease without individual variations. Therefore, pooled data have as a tradeoff lower sensitivity compared to individual data. However, if the numbers of pools are increased, the sensitivity of case-control differences calculated using subpopulation means would increase to the sensitivity calculated using individual data. Ultimately, it is a matter of finding an adequate balance between the number of sample pools and the required precision. To investigate this interdependency more thoroughly, we conducted simulations using data derived from a mathematic model (artificial data), and also applied the methods to data obtained from the measurement of urinary arsenic.

### Simulation sample pooling strategy for case-control comparison artificial data

In order to assess sensitivities, 200 data points each for case and control were generated using either normal or log-normal distributions, according to formulae F2-1, F2-2, F3-1, F3-2, for control normal, case normal, control log-normal, and case log-normal, respectively:

$$x_{\mathrm{control}}=5r\;\left(F2-1\right);\;x_{\mathrm{case}}=m*5r\;\left(F2-2\right)$$

$$x_{\mathrm{control}}=e^{5r}\left(F3-1\right);\;x_{\mathrm{case}}=me^{5r}\left(F3-2\right)$$

**Figure 1:**
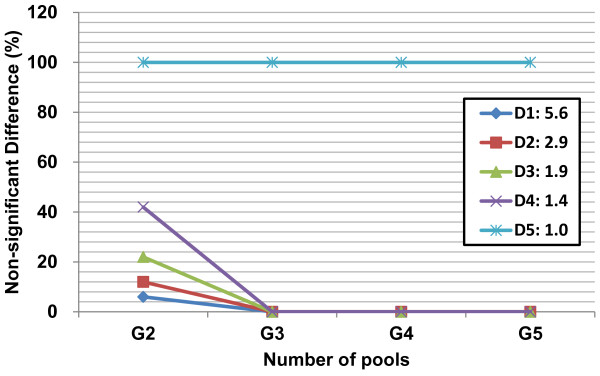
When the random grouping numbers of pools risen to three, the frequency of non-significant difference between the case and control data with normal distributions reached down to the value of that the difference calculated by individual data*. The pool numbers (i.e., numbers of pools in x-axis) was set as 2, 3, 4, and 5 and coded as G2, G3, G4, and G5; 100 times random grouping was applied to the 200 artificial data with normal distributions for the case and control populations, respectively; the means of the case are 1.0, 1.4, 1.9, 2.9 and 5.6-folds of the control and coded as D5, D4, D3, D2 and D1, respectively. *For the upper mentioned normal distribution data, calculated differences by using three random pool means of the case and control, respectively, should be the same as by using the individual data.

**Figure 2:**
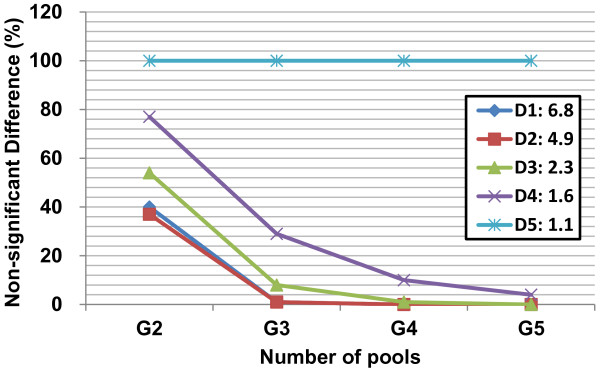
When the random grouping numbers of pools risen to five, the frequency of non-significant difference between the case and control data with log-normal distributions reached down to the value of that the difference calculated by individual data*. The pool numbers (i.e., numbers of pools in x-axis) was set as 2, 3, 4, and 5 and coded as G2, G3, G4, and G5; 100 times random grouping was applied to the 200 artificial data with log-normal distributions for the case and control populations, respectively; the means of the case are 1.1, 1.6, 2.3, 4.9 and 6.8-folds of the control; the means of the case are 1.1, 1.6, 2.3, 4.9 and 6.8-folds of the control and coded as D5, D4, D3, D2 and D1, respectively. *For the upper mentioned log-normal distribution data, calculated differences by using five random pool means of the case and control, respectively, should be close to by using the individual data.

In the simulation models, *r* is a random number between 0 and 1, and *m* is the fold-change between control and case. Although *m* was set to the values 1, 1.5, 2, 3 and 6, the separately random generating of *x*_*control*_ and *x*_*case*_ resulted in the modifications. *m* = 1.0, 1.4, 1.9, 2.9 and 5.6 in the normal distributions (Figure 1), whereas *m* = 1.1, 1.6, 2.3, 4.9 and 6.8 in the log-normal distributions (Figure 2), respectively. Using these generated data, the case-control differences were then tested using the pooled sample means (t-Test with *p* < 0.05 is set for significance), where the numbers of pools were set to 2, 3, 4, and 5 (coded as G2, G3, G4 and G5 in Figures 1 and 2). By performing 100 random regroupings of the two populations, we show that by increasing the number of pools, the frequency of non-significant differences between case and control subpopulations decreases for simulated data with a true difference between case and control (i.e., the set means of the case are *m* = 5.6, 2.9, 1.9 and 1.4-folds of the control in Figure 1 with codes D1, D2, D3 and D4, respectively; the set means of the case are *m* = 6.8, 4.9, 2.3 and 1.6-folds of the control in Figure 2 with codes D1, D2, D3 and D4, respectively). As a result, we conclude that for the normally distributed populations, three or more pooled samples are enough to detect the case-control differences (Figure 1). However, for the log-normally distributed data, a minimum of four sets of pools are required (Figure 2) because the increased number of pools caused their means to be more normally distributed [27]. From the simulated data we conclude that the detection of case-control differences depends on both the difference in the population means, and the number of pools.

### Simulation sample pooling strategy for case-control comparison: measured data

Measured urinary analyte data are often adjusted for creatinine. Accordingly, volume of individual samples used to constitute the sample pool should also be adjusted according to creatinine concentrations. The sample sizes of case-1, case-2 and control are 157, 140 and 151, respectively. Here the case-1 samples consist of urine from subjects with definite idiopathic male infertility and normal semen parameters (unpublished data), the case-2 samples consists of urine from definite idiopathic subjects with male infertility and oligospermia [10] and the control samples of urine from healthy males with normal reproductive function [10]. The measured arsenic species are dimethylarsinic acid (DMA), arsenite (As^III^), methylarsonic acid (MMA), arsenobetaine (AsB) and arsenate (As^V^). Case-1 (CC1 in Table 2) showed FC values of 1.0, 1.1, 1.4, 1.5 and 3.7 compared to control, whereas case-2 (CC2 in Table 2) showed FC values of 1.2, 1.3, 1.2, 1.5 and 3.2 for the analyte DMA, As^III^, MMA, AsB and As^V^, respectively. Statistical analysis showed that the FC versus control calculated by individual observations for As^III^ in case-1, for As^III^, MMA and AsB in case-2 were not robust (i.e. different results were obtained by Welch t-Test and K-S Test), when FC values were ≤ 1.5. However, in some situations, the differences are robust even for FC values close to 1 (DMA in CC1), which may suggest an important role for variance and distribution information in the statistics [26,28]. Moreover, further analysis showed that the dose-dependent adjusted odds ratio trends (Table 2) were only significant for total arsenic along with As^V^ in CC1 (unpublished data) and CC2 [10].

**Table 2:** Descriptive statistics of arsenic species in the case-1, -2, and control subjects:

	**Arsenic in case-1 (n=157)**				**Arsenic in case-2 (n=140)**				**Arsenic in control (n=151)**				**Fold change**		**Welch t-Testt-Testt-Test**		**K-S Test**		**Dose-trend**	
	**Mean**	**SD**	**GM**	**Median**	**Mean**	**SD**	**GM**	**Median**	**Mean**	**SD**	**GM**	**Median**	**CC1**	**CC2**	**CC1**	**CC2**	**CC1**	**CC2**	**CC1**	**CC2**
**Total Arsenic**	246.9	326.2	124.4	140.4	214.1	355.6	96.0	91.4	92.1	172.5	49.6	39.3	2.7	2.3	0.000	0.000	0.000	0.000	Yes	Yes
**AsB**	17.5	29.1	10.5	11.6	17.2	44.6	9.8	9.7	11.4	16.9	8.1	7.9	1.5	1.5	0.026	0.151	0.002	0.004	No	No
**Arsenite(As**^**III**^**)**	4.9	4.1	3.0	4.1	5.7	7.9	3.5	4.1	4.4	3.1	3.4	3.9	1.1	1.3	0.270	0.066	0.034	0.046	No	No
**Arsenate(As**^**V**^**)**	184.9	309.6	44.3	73.3	159.8	343.3	19.6	28.0	50.0	165.0	2.4	1.8	3.7	3.2	0.000	0.001	0.000	0.000	Yes	Yes
**DMA**	23.3	15.9	18.7	19.1	27.1	21.3	20.5	23.4	22.7	15.4	18.2	19.1	1.0	1.2	0.000	0.044	0.000	0.009	No	No
**MMA**	4.9	4.1	3.4	3.9	4.2	3.4	3.1	3.3	3.5	2.5	2.8	2.8	1.4	1.2	0.001	0.073	0.001	0.162	No	No

The case-1, case-2 and control have included the individual samples 157, 140 and 151 in respectively; Welch t-Test: *p*-Value of Welch Modified Two-Sample t-Test; K-S Test: Two-Sample Kolmogorov-Smirnov Test; Dose trend: at least one of the quartiles (the 4^th^ quartile for the monotonic trend) has adjusted odds ratios are significant and the trend analys is  *p* ≤ 0.000 (*χ*^2^ - Test); SD: standard deviation; GM: geometric mean; CC1: control-case-1; CC2: control-case-2.

**Figure 3:**
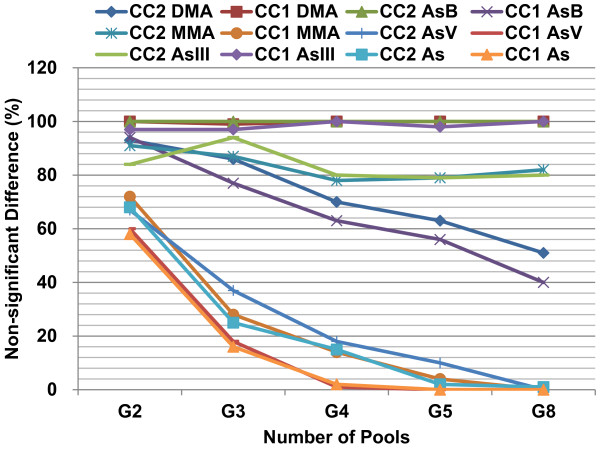
When the random grouping numbers of pools risen to eight, the expected frequency of non-significant difference between the case and control for some arsenic species reached down to the value of that the difference calculated by individual data*. The pool numbers (i.e., numbers of pools in x-axis) was set as 2, 3, 4, 5 and 8 and coded as G2, G3, G4, G5 and G8; n = 157, 140, and 151 for the case-1, case-2 and control subjects (Table 2); 100 times random grouping was applied to the populations; As^V^: arsenate; As: the total arsenic; MMA: methylarsonic acid; As^III^: arsenite; DMA: dimethylarsinic acid; AsB: arsenobetaine. *For the upper mentioned bio-monitoring data can not be normalized by logarithmic transformation, calculated differences by using >5 random pool means of the case and control, respectively, may be close to by using the individual data.

In the present study, simulation was also applied to actual measured arsenic data as previously described. We found that with 100 random groupings of cases and controls, the frequency of non-significant differences between case and control are still high (around 20%) for the ternary groups of the species with FCs ≥2 (Table 2 and Figure 3). For As^V^, MMA (case-1 only), and total arsenic (Figure 3), the pooled sample means show significant difference between case and control, especially when individuals are grouped four or five times. We therefore conclude that pooled subpopulation samples are sufficient to reveal differences between case and control (Figure 3).

In contrast to the artificial log-normally distributed data (Figures 1 and 2), pooled subpopulation means are not sensitive enough to recognize minor, but significant differences between case and control for arsenic for FC ≤1.5 (Table 2). This may be because the data distributions are not exactly log-normal in most situations. However, even if only the subpopulation means are measured, they are still sensitive enough to reveal health risk factors, as shown for As^V^ and total arsenic with FC ≥ 2, which have been linked to the male infertility risk in a dose-dependent manner [10].

### Schematic methodology for exposome analysis

**Figure 4:**
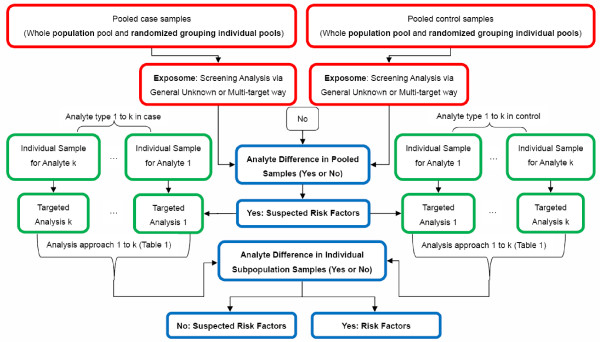
Combing the untargeted and targeted analysis: a scheme for panoramic assessment of the environmental pollutants’ risks in a defined case-control population.

The screened analytes from pooled samples may be further analyzed by utilizing individual samples. However, the volume of individual samples is usually insufficient for broad-spectrum analysis of all pollutants. Instead it is usually necessary to restrict the analysis to one specific class of pollutant in one specific subgroup of samples. For example, urine samples from 30 individual observations can be used for mercapturic acid conjugated pollutant analysis in one subgroup whereas another subgroup can be used to screen for heavy metals (Table 1). Following random selection of the subgroup samples, the statistical parameters of their parental populations can be estimated as well. Finally, we present a methodology workflow (Figure 4) from the pooled sample untargeted screening, to the subgroup individual sample targeted analysis for the molecular exposomein a designed case-control. This method is well suited to meet the challenges presented by exposomics research [29].

## Conclusions

In a traditional molecular epidemiology study (i.e., case-control design), monitoring of individual samples without pooling may prove a sufficient approach. However, in molecular exposomics, a broad-spectrum strategy for hypothesis-free pollutant risk screening, sample pooling is a valuable tool for reducing costs, workload and the need for large individual sample volumes. In this context, the present methodology provides a viable alternative, starting with screening analysis in pooled population samples, followed by randomized grouping of subpopulation samples. Although it has some disadvantages, such as the loss of individual information, it is nonetheless a useful tool. We suggest that approximately five pools and 30 samples per pool from the case and control populations may be sufficient to investigate the disease risk of chemical pollution. However, more sample pools and more samples per pool will always lead to higher quality research.

## Competing interests

The authors declare no competing interests.

## Authors’ contributions

HS and KWS conceived the study; WX measured the urinary arsenic data. HS wrote the draft and SP, HS, JS, KV, AA and KWS helped finish it. All authors read and approved the final manuscript.
